# Factors associated with HIV testing among male injecting drug users: findings from a cross-sectional behavioural and biological survey in Manipur and Nagaland, India

**DOI:** 10.1186/s12954-016-0110-5

**Published:** 2016-06-21

**Authors:** Deepika Ganju, Sowmya Ramesh, Niranjan Saggurti

**Affiliations:** HIV and AIDS Program, Population Council, 142 Golf Links, New Delhi, 110003 India

**Keywords:** Injecting drug users, HIV testing, Counselling, India, Needle and syringe, Nagaland, Manipur

## Abstract

**Background:**

Although targeted interventions in India require all high-risk groups, including injecting drug users (IDUs), to test for HIV every 6 months, testing uptake among IDUs remains far from universal*.* Our study estimates the proportion of IDUs who have taken an HIV test and identifies the factors associated with HIV testing uptake in Nagaland and Manipur, two high HIV prevalence states in India where the epidemic is driven by injecting drug use.

**Methods:**

Data are drawn from the cross-sectional Integrated Behavioural and Biological Assessment (2009) of 1650 male IDUs from two districts each of Manipur and Nagaland. Participants were recruited using respondent-driven sampling (RDS). Descriptive data were analysed using RDSAT 7.1. Multivariate logistic regression analysis was undertaken using STATA 11 to examine the association between HIV testing and socio-demographic, behavioural and programme exposure variables.

**Results:**

One third of IDUs reported prior HIV testing, of whom 8 % had tested HIV-positive. Among those without prior testing, 6.2 % tested HIV-positive in the current survey. IDUs aged 25–34 years (adjusted odds ratio (OR) = 1.41; 95 % confidence interval (CI) = 1.03–1.93), married (Adjusted OR = 1.56; 95 % CI = 1.15–2.12), had a paid sexual partner (Adjusted OR = 1.64; 95 % CI = 1.24–2.18), injected drugs for more than 36 months (Adjusted OR = 1.38; 95 % CI = 1.06–1.81), injected frequently (Adjusted OR = 1.49; 95 % CI = 1.12–1.98) and had high-risk perception (Adjusted OR = 1.68; 95 % CI = 1.32–2.14) were more likely than others to test for HIV. Compared to those with no programme exposure, IDUs who received counselling, or counselling and needle/syringe services, were more likely to test for HIV.

**Conclusions:**

HIV testing uptake among IDUs is low in Manipur and Nagaland, and a critical group of HIV-positive IDUs who have never tested for HIV are being missed by current programmes. This study identifies key sub-groups—including early initiators, short duration and less frequent injectors, perceived to be at low risk—for promoting HIV testing. Providing needles/syringes alone is not adequate to increase HIV testing; additionally, interventions must provide counselling services to inform all IDUs about HIV testing benefits, facilitate visits to testing centres and link those testing positive to timely treatment and care.

## Background

Injection drug users (IDUs) are a group at high risk of acquiring and transmitting HIV due to unsafe injection and sexual practices [1–3]. Although IDUs are a priority group for targeted interventions in India where the epidemic is concentrated among high-risk groups, rising HIV prevalence among IDUs continues to be a concern. Nationally, HIV prevalence (2014–2015) is higher among IDUs (9.9 %) than female sex workers (FSWs) and men who have sex with men (MSM) (2.2 and 4.3 % respectively), and while the epidemic has been stabilising among FSWs and MSM, HIV prevalence among IDUs has been rising [4]. Moreover, new IDU-driven epidemics are emerging in India, with several states/regions reporting high HIV prevalence (>10 %) among IDUs [4].

HIV testing is a cost-effective strategy for the reduction of HIV risk and transmission [5] and provides an entry point for prevention, treatment and care. Early HIV diagnosis can lead to timely initiation of treatment [6–8] and knowledge of sero-positive status through testing can lead to the adoption of safer injection and sexual behaviours [2, 9–13]. Additionally, HIV testing provides a critical opportunity to counsel clients on risk reduction. HIV testing is a priority of the Indian government, and targeted interventions require all high-risk groups, including IDUs, to test for HIV once every 6 months [14, 15]. With the aim of increasing HIV testing accessibility and availability, the Indian government has scaled up voluntary counselling and testing (VCT) services for HIV across the country [15].

In India, the majority of IDUs are from the states of Manipur and Nagaland, two high HIV prevalence states where the HIV epidemic is primarily driven by injection drug use [6] [5]. The vast majority of IDUs in Manipur and Nagaland are male [16], and approximately 2 % of the adult population in these states engage in injecting drugs, mainly heroin and spasmo-proxyvon (a synthetic opioid analgesic) [17]. These mountainous states are located on India’s north-eastern border with Myanmar, where illicit drugs are readily accessible [18]. These states have primarily rural populations and are characterised by ethnic and linguistic diversity; poor road and health service infrastructure; ethnic conflict; armed insurgency; poverty; and unemployment [16]. These two states have historically been priority states for targeted IDU interventions under the Indian government’s National AIDS Control Programme (NACP). Additionally, Avahan, the India AIDS initiative has been implementing a scaled up comprehensive harm reduction programme (2004–2014) for IDUs in these states. Services include distribution of sterile needles and syringes, counselling, condom distribution, sexually transmitted infection (STI) treatment, abscess management, opioid substitution therapy (OST), VCT referrals and linkages to detoxification/rehabilitation services [19, 16]. Although IDUs have been the focus of targeted services in these two states, infection rates among IDUs continue to be high (12.1 % in Manipur and 3.2 % in Nagaland, 2014–2015 estimates) [4]. Manipur and Nagaland were among the states that recorded the highest HIV prevalence (0.64 and 0.88 %, respectively; 2012–2013 estimates) among antenatal clinic attendees [20], and adult HIV prevalence in Manipur and Nagaland (1.22 and 0.73 %; 2011 estimates) was higher than the national prevalence rate (0.27 %) [21].

Although prevention interventions in India have focused on saturating HIV testing among high-risk groups, including IDUs, testing uptake among IDUs remains far from universal [4]. As a result, many IDUs who are HIV infected are unaware of their status and may transmit the virus to others. Despite the critical importance of IDUs to HIV prevention and care efforts, limited information is available on the characteristics of IDUs undergoing HIV testing in India. To address this gap, our study estimates the proportion of IDUs who have taken an HIV test and identifies the factors associated with HIV testing uptake in Nagaland and Manipur, two high HIV prevalence states in India where the epidemic is driven by injecting drug use. Our findings will provide valuable inputs for future programmes aimed at increasing HIV testing uptake and reducing HIV transmission, among IDUs in India.

## Methods

### Study design

Data for this study are drawn from the 2009 Integrated Behavioural and Biological Assessment (IBBA), a cross-sectional survey that used an interviewer-administered questionnaire and the collection of blood samples to test for HIV. The survey was conducted in April–June among male IDUs in two districts of Manipur (Churachandpur and Bishnupur) and in May–July in two districts of Nagaland (Phek and Wokha). Approval for the study protocol was obtained from the Government of India’s Health Ministry Screening Committee and the ethical review boards of participating institutions. A comprehensive consent process was adopted: respondents were informed about all aspects of the survey, following which verbal consent was obtained individually for the behavioural and biological component. Participation was voluntary with the option to withdraw at any time. Questionnaires and biological testing were anonymously linked so the results could not be traced back to the respondent.

### Sampling

Participants were recruited using respondent-driven sampling (RDS). RDS is a validated probability sampling method based on conventional sampling used to recruit hidden populations such as sex workers and IDUs [22, 23]. For this study, four to eight seeds from the target population who met the study eligibility criteria were purposively selected to reflect the diversity of demographic characteristics. Each seed participant was given three uniquely coded coupons to refer three eligible IDU peers from his network for recruitment into the study. These recruits, in turn, were provided three coupons each to recruit three additional IDUs for the next wave. This peer-to-peer recruitment process continued until the desired sample size was achieved. All participants were provided a primary incentive (INR 70; 1 USD = approximately INR 65) for participating in the study and a secondary incentive (INR 30) for recruiting an eligible peer into the study [24].

In this study, a sample size of 400 was estimated for each survey district, which allowed for detection of an absolute difference of 15 % or more from the assumed value of 50 % with 95 % confidence (5 % probability of type I error) and 90 % power (10 % probability of type II error), for indicators such as use of clean needles. A design effect of 1.5 was applied. Methodological details of the study have been described elsewhere [14].

### Data collection

Eligible individuals were male, aged 18 years or older, who had injected drugs for non-medical purposes at least once in the last 6 months. Face-to-face interviews were conducted by trained investigators in the local language, using a structured questionnaire that included questions on socio-demographic characteristics, injecting behaviour, sexual behaviour, exposure to interventions and prior HIV testing. Additionally, respondents provided finger prick blood samples for HIV testing [14].

### Statistical analysis

Descriptive data were analysed using RDSAT 7.1 to generate adjusted proportion estimates with 95 % confidence intervals. Multivariate logistic regression analysis was undertaken using STATA 11 to examine the association between HIV testing prior to the survey and the explanatory variables (age, marital status, occupation, paid sexual partner, regular sexual partner, duration of injection use, frequency of injecting drugs, sharing needles/syringes, knowledge of STI, risk perception and programme exposure). Results are presented for the overall sample as the site-specific associations were observed to be generally similar. Individualised weights were generated for the dependent variable (i.e. ever tested for HIV) using RDSAT analysis and applied to the logistic regression analysis in STATA.

### Measures

The dependent measure considered in this study was ever tested for HIV (yes/no). Current HIV status was determined based on the laboratory test result, and respondents were considered HIV-positive if their blood samples tested positive on the Microlisa test and confirmed by the Genedia HIV ½ ELISA 3.0 test.

Socio-demographic characteristics included age, occupation and marital status. Age was measured as a continuous variable and grouped into three categories (18–24; 25–34; 35 years and above). Marital status was categorised as being never married or ever married, and employment status recorded as a response to a direct question and grouped into three categories: unemployed, student and employed.

The sexual behaviours considered in the study include had a regular sex partner ever (yes/no) and had a paid sex partner ever (yes/no). The injection practices considered in this study include duration of injection use, frequency of injection in the past 1 month and sharing needles/syringes (yes/no) in the past 1 year. Duration of injection use was measured as a continuous variable and categorised into two groups (injecting drugs for 36 months or less/injecting drugs for more than 36 months). Frequency of injection use was dichotomised as less frequent (defined as injecting three times or less in the past month) or more frequent (defined as injecting more than three times in the past month).

Other key independent variables considered include knowledge of STI (Have you ever heard of diseases that can be transmitted through sexual intercourse (also known as STI)? (yes/no)); risk perception (Do you feel that you are at risk for becoming infected with HIV/AIDS?) (high risk/low risk)); and programme exposure. Participants were divided into three groups to assess programme exposure in the past 6 months: those who received no programme services were coded as 1 (no exposure); those who received only needle and syringe services were coded as 2; and those who received only counselling services from a peer educator/outreach worker or both counselling from a peer educator/outreach worker and needle and syringe services were coded as 3.

## Results

### Profile of respondents

A total of 1650 IDUs (Manipur, *N* = 821; Nagaland, *N* = 829) were recruited (Table 1). State-wise differences were noted in age distribution and marital status. Overall, the majority were in the age group 25–34 years; nearly half (46 %) were employed and three-fifths (60 %) were never married. Most respondents (59 %) had a regular sexual partner and one fifth (20 %) had a paid sexual partner. Differences by state were observed with regard to sexual partners: a higher proportion of IDUs from Nagaland than Manipur reported ever having a regular sexual partner whereas a higher proportion of IDUs from Manipur as compared to Nagaland reported ever having had a paid sexual partner.

**Table 1:** Profile of IDUs, Manipur and Nagaland and overall

Characteristics	Manipur % (95 % CI)	Nagaland % (95 % CI)	Overall % (95 % CI)
Age (years)			
18–24	22.6 (18.5–26.7)	44.6 (39.3–49.4)	33.2 (29.7–36.5)
25–34	59.9 (55.4–64.4)	40.9 (36.1–46.0)	50.6 (47.2–54.0)
35+	17.5 (14.2–21.1)	14.5 (11.2–18.2)	16.2(13.9–18.7)
Occupation			
Unemployed	42.3 (37.8–47.2)	49.7 (4.43–5.50)	46.2 (4.26–4.97)
Student	3.9 (2.0–6.4)	13.3 (9.9–17.7)	8.4 (6.5–10.7)
Employed	53.7 (48.8–58.3)	37.0 (31.9–41.6)	45.5 (42.1–48.8)
Marital status			
Never married	53.7 (48.8–58.4)	65.2 (60.4–69.4)	59.6 (56.2–62.9)
Ever married	46.3 (41.6–51.2)	34.8 (30.6–39.6)	40.4 (37.1–43.8)
Sexual practices			
Had a regular sexual partner ever^a^			
No	55.8 (51.2–60. 2)	27.0 (22. 6–31. 8)	41.5 (38. 0–44.9)
Yes	44.2 (39.8–48.8)	73.0 (68.2–77.4)	58.5 (55.1–62.0)
Had a paid sexual partner ever^b^			
No	69.1 (64.8–73.3)	90.8 (87.8–93.2)	80.2 (77.5–82.9)
Yes	30.9 (26.7–35.2)	9.2 (6.8–12.2)	19.8 (17.1–22.5)
Injecting practices			
Duration of injecting drug use (months)			
<=36	34.9 (30.3–40.2)	69.6 (65.0–73.6)	51.9 (48.5–55.5)
>36	65.1 (59.8–69.7)	30.4 (26.4–35.0)	48.1 (44.5–51.5)
Frequency of injecting drugs in past 1 month^c^			
Less frequent	25.1 (20.5–30.2)	44.3 (39.4–49.1)	34.1 (30.6–37.3)
More frequent	74.9 (69.8–79.5)	55.7 (50.9–76.0)	65.9 (6.27–6.94)
Shared needles/syringes in past 1 year			
No	43.4 (38.3–48.2)	39.6 (35.2–44.6)	41.3 (38.0–44.7)
Yes	56.6 (51.8–61.7)	60.4 (55.4–64.8)	58.7 (55.3–62.0)
Knowledge of STI			
Not aware	22.7 (18.4–26.8)	31.7 (26.6–36.5)	26.8 (23.7–30.3)
Aware	77.3 (73.2–81.6)	68.3 (63.5–73.4)	73.2(69.7–76.3)
Risk perception			
Low risk	50.3 (45.5–54.9)	68.7 (64.3–73.2)	59.7 (56.6–63.2)
High risk	49.7 (45.1–54.5)	31.3 (26.8–35.7)	40.3 (36.8–43.4)
Ever taken an HIV test prior to survey			
No	52.4 (47.6–56.7)	83.0 (79.4–85.9)	67.4 (64.5–70.5)
Yes	47.6 (43.3–52.4)	17.0 (14.1–20.6)	32.6 (29.5–35.6)
HIV status			
HIV-negative	72.3 (67.9–76.5)	98.5 (97.3–99.5)	84.4 (80.8–86.4)
HIV-positive	27.7 (23.5–32.1)	1.5 (0.5–2.7)	15.6 (13.6–19.2)
Programme exposure (last 6 months)			
No exposure	18.7 (14.4–23.4)	55.0 (48.7–60.8)	33.6 (29.1–36.8)
Received only needle syringe services	27.3 (23.9–31.6)	12.2 (9.3–15.3)	20.2 (17.5–22.8)
Received only counselling services or counselling and needle/syringe services	54.0 (48.8–58.4)	32.8 (27.9–38.4)	46.2 (43.4–50.7)

*CI* confidence interval, *STI* sexually transmitted infection

^a^Regular sexual partner: main steady partner/spouse with whom IDUs had sex ever

^b^Paid sexual partner: women with whom IDUs had sex ever in exchange for cash/drugs/kind ever

^c^Less frequent includes those who injected drugs three times or less in the past 1 month; more frequent includes those who injected drugs more than three times in the past 1 month

Two thirds of IDUs (66 %) reported injecting frequently (more than three times in the past month), and almost half (48 %) reported long-term injecting use (more than 36 months). Significant differences were observed across states in terms of duration of injecting drug use, with a higher proportion of IDUs in Manipur as compared to Nagaland injecting for more than 36 months. More than half (59 %) of the IDUs had shared needles/syringes in the past 1 year. Almost three fourths (73 %) were aware of STIs, and two fifths perceived themselves to be at high HIV risk.

Around one third (34 %) of IDUs had no programme exposure in the past 6 months; while two fifths (20 %) had received only needles and syringes from the programme, almost half (46 %) had received only counselling services or counselling and needle/syringe services from the programme.

One third (33 %) of IDUs reported ever having undergone a prior HIV test; of these, 8 % had tested HIV-positive. Significant differences were noted across the states, with a higher proportion of IDUs from Manipur than Nagaland ever having taken an HIV test. Of those who reported prior testing, a higher proportion of IDUs from Manipur (28 %) than Nagaland (2 %) had tested HIV-positive. Among those who had not undergone prior HIV testing, 6.2 % tested positive in the current survey (not shown in tabular form).

### Factors associated with prior HIV testing

As seen in Table 2, IDUs in the age group 25–34 years (adjusted odds ratio (OR) = 1.41; 95 % confidence interval (CI) = 1.03–1.93)) and who were ever married (Adjusted OR = 1.56; 95 % CI = 1.15–2.12) were more likely than their counterparts to test for HIV. IDUs who had a paid sexual partner (Adjusted OR = 1.64; 95 % CI = 1.24–2.18) and those who had injected drugs for more than 36 months were more likely than others to test for HIV (Adjusted OR = 1.38; 95 % CI = 1.06–1.81). Similarly, IDUs who injected more frequently (Adjusted OR = 1.49; 95 % CI = 1.12–1.98) had a higher likelihood of testing for HIV compared to those who injected less frequently. IDUs who had high-risk perception (Adjusted OR = 1.68; 95 % CI = 1.32–2.14) were more likely to test for HIV compared to their counterparts. Additionally, compared to those with no programme exposure, IDUs who reported either receiving only peer counselling, or both peer counselling and needle/syringe services from the programme in the past 6 months, were more likely to test for HIV.

**Table 2:** Factors associated with HIV testing among IDUs, Manipur and Nagaland, overall

Variables	% (*N* = 652)	Overall adjusted OR (95 % CI)
Age (years)		
18–24	26.75 (126)	Ref
25–34	45.13 (408)	1.41 (1.03–1.93)
35+	42.91 (118)	1.07 (0.69–1.66)
Occupation		
Unemployed	36.53 (255)	Ref
Student	32.06 (42)	1.27 (0.80–2.02)
Employed	43.24 (355)	1.23 (0.96–1.59)
Marital status		
Never married	34.18 (324)	Ref
Ever married	46.72 (328)	1.56 (1.15–2.12)
Had a regular sexual partner ever^a^		
No	39.45 (258)	Ref
Yes	39.56 (394)	0.96 (0.74–1.25)
Had a paid sexual partner ever^b^		
No	35.49 (456)	Ref
Yes	53.70 (196)	1.64 (1.24–2.18)
Duration of injecting drug use (months)		
<=36	26.68 (203)	Ref
>36	50.51 (449)	1.38 (1.06–1.81)
Frequency of injecting drugs in past month^c^		
Less frequent	25.15 (127)	Ref
More frequent	45.85 (525)	1.49 (1.12–1.98)
Shared needles/syringes in past year		
No	38.51 (223)	Ref
Yes	40.06 (429)	1.08 (0.84–1.38)
Knowledge of STI		
Not aware	18.13 (68)	Ref
Aware	45.80 (584)	1.39 (0.10–1.93)
Risk perception		
Low risk	28.68 (255)	Ref
High risk	52.17 (397)	1.68 (1.32–2.14)
Programme exposure (last 6 months)		
No exposure	12.47 (61)	Ref
Received only needle/syringe services	33.64 (110)	1.05 (0.48–2.32)
Received only counselling services or counselling and needle/syringe services	57.67 (481)	2.44 (1.06–5.60)

Model controlled for the variables considered in the analysis

*OR* odds ratio, *CI* confidence interval, *STI* sexually transmitted infection

^a^Regular sexual partner: main steady partner/spouse with whom IDUs had sex ever

^b^Paid sexual partner: women with whom IDUs had sex ever in exchange for cash/drugs/kind ever

^c^Less frequent includes those who injected drugs three times or less in the past 1 month; more frequent includes those who injected drugs more than three times in the past 1 month

## Discussion

Despite the scale up of VCT services in India, our study shows that HIV testing rates among IDUs are low in Manipur and Nagaland, where the HIV epidemic is driven by injection drug use. Although these states have been prioritised for IDU-focused HIV prevention programmes, just one third of male IDUs in these settings had ever tested for HIV despite 66 % having accessed programme services; these rates are far below the national goal of saturating HIV testing among high-risk groups in India [15]. Similar low testing rates among IDUs have been reported in other studies in India as well [25–27].

Under targeted interventions, all high-risk groups, including IDUs, must test for HIV every 6 months; however, recent estimates (2014–2015) indicate that testing rates remains far below the desired level. One third of IDUs nationally have never tested for HIV, and in some high HIV prevalence regions/states such as Bihar and Uttar Pradesh (HIV prevalence among IDUs >27 %), less than one third had taken an HIV test ever [4]. Although nationally ever-testing rates among IDUs have increased from those reported in our study, perhaps as a result of focused interventions for IDUs under NACP, the continued testing gap is a challenge for HIV prevention efforts, particularly in the context of IDUs’ high-risk behaviours, rising HIV prevalence in this group and emerging IDU-driven epidemics in several states/regions [4]. Notably, in India, nationally, HIV ever-testing rates are lowest among IDUs as compared to MSM (78 %) and FSWs (84 %) [4]. Increased prevention efforts for IDUs, including regular VCT uptake by all IDUs across all states in the country is crucial, given the potential for HIV transmission in this high-risk group.

State-level differences in HIV testing are observed; far fewer IDUs in Nagaland than Manipur reported ever taking an HIV test, which may in part be explained by lower self-perceived HIV risk in Nagaland, Nagaland’s relatively more difficult terrain, hindering access to testing facilities [28], and Manipur’s historically higher HIV prevalence among IDUs, resulting in a more effective testing response [27]. Despite these differences in testing rates, IDUs in both states have high HIV prevalence and should continue to be the focus of HIV prevention programmes.

Of concern is the finding that among IDUs without prior HIV testing, around 6 % tested HIV-positive in the current study. These IDUs are most at risk, as they are unaware of their sero-status and, therefore, may not be linked to treatment and may continue to transmit HIV infection through their risk behaviours [2, 26]. As early detection is essential to provide timely care and treatment, and prevent further HIV transmission, programmes must ensure that all IDUs test regularly for HIV, promote the adoption of risk reduction behaviours and link HIV-positive IDUs to timely care and treatment.

Our study identifies several sub-groups of drug injectors who are less likely to test for HIV and hence at high risk of acquiring and transmitting HIV; these critical groups should be the focus of intervention programmes to promote HIV testing. While less frequent injectors and recent injectors are considered to be at lower risk [3, 26, 29], our findings support published studies documenting the HIV vulnerability of new and recent initiators and less frequent injectors [30–33] as they are less likely than their counterparts to take an HIV test. Corroborating these findings, a post hoc analysis indicates that around two thirds of IDUs in this study who shared injection equipment are short-term injectors and less frequent injectors.

While studies have documented that IDUs’ risky injection and sexual behaviours elevate the risk of HIV transmission to their sexual partners [3, 31, 34], our study reports that IDUs with paid sex partners, a particularly high-risk group, are more likely than others to test for HIV, suggesting that targeted interventions have been effective in promoting protective behaviours. At the same time, the finding that most IDU respondents had a regular sexual partner, but the odds of testing were not higher in this group, suggests that this sub-group of IDUs and their sexual partners are at risk of acquiring and transmitting HIV [1, 3, 25]. Our study also highlights the vulnerability of unmarried IDUs [3] as they are less likely than married IDUs to test for HIV. As HIV counselling and testing promote safe sex behaviours among IDU populations [1, 35], efforts are needed to encourage all IDUs who are sexually active to test regularly for HIV, to prevent the onward transmission of infection to their sexual partners.

Consistent with other studies, our study shows that high perceived risk of HIV infection is a significant predictor for HIV testing [36, 37]. Given the evidence that approximately three fifths of IDUs in our study perceived they were at low HIV risk, despite their risky behaviours, efforts to increase HIV testing among IDUs must strengthen strategies to build awareness of personal risk.

While our study findings indicate that programme exposure is positively associated with HIV testing, not all IDUs reported recent contact with the programme. Notably, IDUs who received counselling were far more likely to test for HIV than other IDUs—those without recent programme contact or those who received only clean injection equipment from the programme. These findings suggest that the utilisation of needle and syringe services alone does not necessarily translate into testing uptake and must be supported with focused risk reduction information for sustained behaviour change [28] to maximise HIV testing.

Prior research among high-risk groups has documented that multiple factors influence the decision to undergo HIV testing. Individual-level barriers, including anxiety about a positive test result, lack of awareness about antiretroviral treatment (ART), low HIV risk perception and fear of being seen accessing HIV services, as well as structural barriers, such as HIV-related stigma, criminalisation of drug use, quality of care (discrimination by health providers) and accessibility issues (inconvenient testing hours, travel cost, long registration process and long waiting time to receive test result) [38, 39] need to be addressed if HIV testing uptake among IDUs is to be maximised.

Peer-led outreach can expand programme coverage among marginalised groups and address HIV testing barriers. Peers are often the initial source of information on HIV, including testing benefits and available testing and treatment facilities [40–43]; they can reach hidden IDUs and can be trusted to keep HIV status confidential [38, 39]. Moreover, peers can accompany IDUs to health testing facilities if necessary and sensitise health workers to IDUs’ special needs. Prevention programmes must invest in strengthening existing peer networks to increase HIV testing uptake. Peers should be given well-defined targets, including for referrals to VCT centres, to maximise testing.

The study findings also point to the need for programmes to strategically focus on counselling, in addition to providing needle and syringe services, to promote HIV testing. Counselling sessions must build awareness of the benefits of regular HIV screening and early HIV diagnosis and treatment; promote awareness of the efficacy of ART and the availability of government-supported treatment services; and raise the perception of personal risk for HIV. Repeated and time-intensive peer counselling is more effective than single sessions in reinforcing behaviour change [41] and should be adopted by the programme.

Additionally, integrating HIV testing at the primary healthcare level is a cost-effective strategy to increase HIV screening, provide linkages to ART centres and reduce HIV testing stigma [8, 44]. Voluntary drug treatment facilities are well-suited to promoting HIV testing; they provide IDUs an important point of contact with the health system and can engage individuals in HIV testing by linking them to testing centres; moreover, drug users who come for treatment may be ready to initiate behaviour change [39, 45, 46]; the OST programme for IDUs, which is being scaled up in India [47], could play an important role in promoting testing coverage across the country. Mobile HIV testing clinics could increase testing accessibility in remote areas [18]. New and short-term injectors, who are less likely to test for HIV, can be reached by enlisting young peer educators and organising social activities to attract them to testing services [18]. Youth clubs, which have wide reach in the country, could be engaged to reach young IDUs and create awareness about injection risk behaviours and promote HIV testing. These pilots could be scaled up if successful.

While this study provides important insights to promote HIV testing among IDUs to prevent further HIV transmission in this high-risk group, the findings must be interpreted in light of certain limitations. First, the study is based on self-reported risk behaviours, which may be subject to social desirability bias, and as a result, socially unacceptable behaviours may have been underreported. The use of trained field staff may have increased participants’ level of comfort at the time of interview and reduced underreporting. We considered only male IDU respondents for the current analysis, and the study may have missed a group of female IDUs who have not tested for HIV. Anecdotal evidence suggests that non-injecting drug use is more common among female drug users [26], and given the evidence that the vast majority of IDUs in India are male, we limited the current analysis to male IDUs only. Further, as injecting drug use characteristics vary across states in India, our results may not be generalisable to IDUs in other states and settings. However, these limitations do not compromise the internal validity of the data.

## Conclusions

HIV testing uptake is low among IDUs in Manipur and Nagaland, India. A critical group of IDUs—those who are HIV-positive but have not taken an HIV test and are unaware of their HIV status—need urgent programme focus to build awareness of their HIV status and link them to timely treatment and care. HIV prevention programmes must intensify efforts to reach all IDUs, focusing on critical sub-groups, including early initiators, short duration and less frequent injectors, perceived to be at low risk, to promote regular HIV testing and other risk reduction behaviours. Providing needles/syringes alone is not adequate to increase HIV testing; additionally, interventions must focus on providing counselling services to inform all IDUs about HIV testing benefits, facilitate visits to testing centres and link HIV-positive persons to timely treatment and care.

## Abbreviations

ART, antiretroviral treatment; CI, confidence interval; ELISA, enzyme-linked immunosorbent assay; FSW, female sex worker; HIV, human immunodeficiency virus; IDU, injecting drug user; INR, Indian rupee; MSM, men who have sex with men; NACP, National AIDS Control Programme; OR, odds ratio; OST, opioid substitution therapy; RDS, respondent-driven sampling; STI, sexually transmitted infection; USD, US dollar; VCT, voluntary counselling and testing
